# Myeloid-Derived Suppressor Cells as Therapeutic Target in Hematological Malignancies

**DOI:** 10.3389/fonc.2014.00349

**Published:** 2014-12-08

**Authors:** Kim De Veirman, Els Van Valckenborgh, Qods Lahmar, Xenia Geeraerts, Elke De Bruyne, Eline Menu, Ivan Van Riet, Karin Vanderkerken, Jo A. Van Ginderachter

**Affiliations:** ^1^Laboratory of Hematology and Immunology, Myeloma Center Brussels, Vrije Universiteit Brussel, Brussels, Belgium; ^2^Laboratory of Cellular and Molecular Immunology, Vrije Universiteit Brussel, Brussels, Belgium; ^3^Laboratory of Myeloid Cell Immunology, VIB, Brussels, Belgium

**Keywords:** myeloid-derived suppressor cells, immune system, hematological malignancies, multiple myeloma, leukemia, lymphoma, stem cell transplantations

## Abstract

Myeloid-derived suppressor cells (MDSC) are a heterogeneous population of immature myeloid cells that accumulate during pathological conditions such as cancer and are associated with a poor clinical outcome. MDSC expansion hampers the host anti-tumor immune response by inhibition of T cell proliferation, cytokine secretion, and recruitment of regulatory T cells. In addition, MDSC exert non-immunological functions including the promotion of angiogenesis, tumor invasion, and metastasis. Recent years, MDSC are considered as a potential target in solid tumors and hematological malignancies to enhance the effects of currently used immune modulating agents. This review focuses on the characteristics, distribution, functions, cell–cell interactions, and targeting of MDSC in hematological malignancies including multiple myeloma, lymphoma, and leukemia.

## Introduction

**Figure 1:**
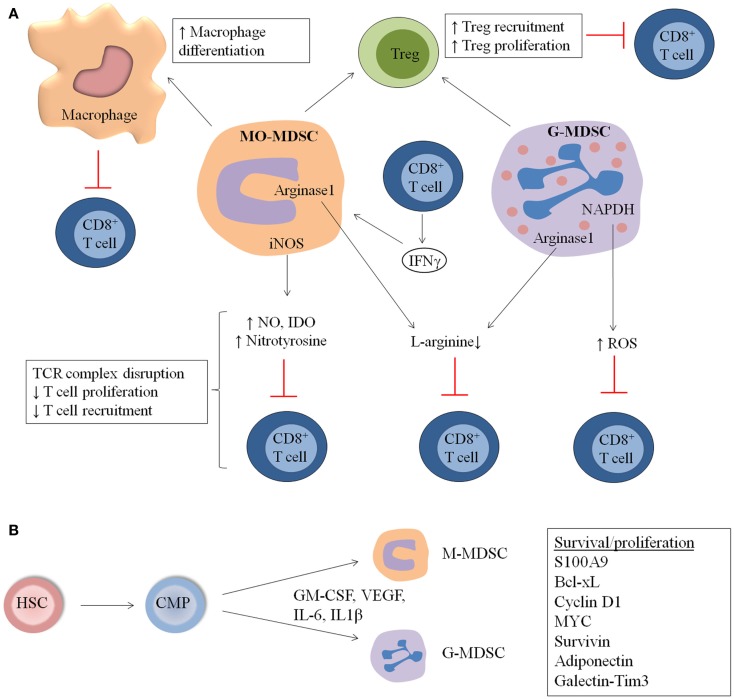
**General overview of MDSC immunosuppressive mechanisms and expansion in hematological malignancies and during stem cell transplantation (SCT)**. **(A)** MDSC suppress the immune system by distinct mechanisms including increased macrophage differentiation and regulatory T cell (Treg) proliferation, direct actions of MDSC on T cells by increased NO, nitrotyrosine and ROS secretion, and decreased l-arginine production. **(B)** MDSC originate from common myeloid progenitors (CMP), which arise from hematopoeitic stem cells (HSC). M-MDSC and G-MDSC are formed and proliferate in the presence of distinct factors including GM-CSF, IL-6, VEGF, and IL-1β. Factors involved in the proliferation and survival of MDSC are S100A9, Cyclin D1, Bcl-xL Myc, and Survivin.

It has been widely accepted that the bone marrow (BM) microenvironment becomes immunosuppressive and plays a crucial role in cancer development and progression (1, 2). Immune suppression is caused by inhibition of activated immune cells, and generation or expansion of immunosuppressive cell types. Regulatory T cells, myeloid-derived suppressor cells (MDSC) and tumor-associated macrophages (TAM) all contribute to an immunologically permissive microenvironment for cancer cells (2–4). MDSC are generally defined as a heterogeneous cell population that arises from myeloid progenitor cells in the BM. These progenitor cells have the capacity to differentiate into macrophages, dendritic cells (DC), or granulocytes, or remain in an undifferentiated state (defined as MDSC) (5, 6). In healthy mice, immature myeloid cells are present in the BM and in small numbers in the spleen; however, they highly accumulate in spleen, lymph nodes, and tumor tissue of tumor-bearing mice (6–8). MDSC are increased in solid tumors (e.g., breast cancer, colorectal cancer) as well as hematological malignancies [e.g., multiple myeloma (MM), leukemia, and lymphoma] (9–14). MDSC expansion and activation is promoted by tumor cells as well as their microenvironment, mainly by the secretion of cytokines and growth factors (8). For mice, MDSC are characterized based on dual expression of CD11b and GR1. Furthermore, MDSC are subdivided into Ly6G^low^ (monocyte morphology, MO-MDSC) and Ly6G^high^ cells (polymorphonuclear morphology, PMN-MDSC, or G-MDSC) (5, 6, 15). Human MDSC express the myeloid markers CD11b and CD33. CD14^+^HLA-DR^low/−^ MDSC are mainly described as the monocytic subpopulation, while human granulocytic MDSC are mostly defined to be CD15^+^ (16). MDSC inhibit innate and adaptive immunity by regulatory T cell activation and secretion of nitric oxide (NO), reactive oxygen species (ROS), and immunosuppressive cytokines (e.g., IL-10) (6, 17). They not only affect the immune system but also promote tumor angiogenesis, tumor cell invasion, and metastasis. A more extensive description of the phenotype and functional mechanisms of MDSC in general is available in previous reviews (8, 18–20). In the last years, efforts have been made to unravel the characteristics, distribution, and function of murine and human MDSC in hematological malignancies as illustrated in Table 1 and Figure 1. This will be summarized in this review.

**Table 1:** **General overview of MDSC phenotype**.

Species	Disease/condition	Markers	MDSC subtypes	Reference
Murine	MM	CD11b^+^GR1^+^	Ly6G^low^ or MO-MDSC	(10, 21, 22)
	Lymphoma		Ly6G^high^ or G-MDSC	
	Leukemia	
Human	MM	CD11b^+^CD33^+^	CD14^+^HLA-DR^−/low^ or MO-MDSC	(23)
			CD14^−^CD15^+^ or G-MDSC	(12, 22)
	Lymphoma	–	CD14^+^HLA-DR^low^	(14)
	Leukemia	CD11b^+^CD33^+^	CD14^−^HLA-DR (AML)	(24)
			CD14^−^arginase-1^+^ (CML)	(25)
			CD14^+^HLA-DR^low^ (CLL)	(13)
	Allo-HSCT	–	CD14^+^HLA-DR^low^	(26)

*Markers described to characterize murine and human MDSC in multiple myeloma (MM), lymphoma, leukemia and allogeneic hematopoietic stem cell transplantation (Allo-HSCT)*.

## MDSC in Multiple Myeloma

Multiple myeloma is a malignant plasma cell disorder characterized by the accumulation of neoplastic plasma cells in the BM and the presence of monoclonal immunoglobulins in the blood and/or urine. Clinical features of this disease include anemia, bone pain, renal failure, frequent occurrence of infections, and hypercalcemia (27). A hallmark of MM is the reciprocal interaction between tumor cells and the BM microenvironment, resulting in accelerating bone loss, increased blood vessel formation, and progressive cancer growth (28). MM is the second most common hematological malignancy and constitutes 1% of all cancers and 13% of all hematological cancers. It affects mostly elderly patients (the median age of diagnosis is 67 years), though 3% of all MM patients are under the age of 40 years. The survival of patients has been improved by new agents like the proteasome inhibitor bortezomib and the immune modulating drug lenalidomide in combination with the alkylating agent melphalan and dexamethasone (29). Despite this evolution, patients still relapse or become refractory to treatment, indicating the need for new therapies.

### Immune dysfunction in MM

Immune dysfunction is an important feature of MM patients and leads to infections and increased tumor growth. A variety of immune defects are observed in MM including cellular abnormalities (e.g., B cells, T cells, DC), secretion of immunosuppressive cytokines (e.g., TGF-β, VEGF, HGF), and increased frequencies of immunosuppressive cell types (including regulatory T cells and MDSC) (30, 31). B-cells derived from MM patients showed impaired immunoglobulin synthesis and secretion (32). Therefore, intravenous administration of immunoglobulin has been used to restore the antibody-mediated immunity in patients with hypogammaglobulinemia (33). Besides B-cell defects, the T cell-mediated immunity is also impaired in MM patients. CD4^+^ T cells and in particular T-helper 2 cells (Th2) were significantly decreased in MM patients, while no difference in CD8^+^ T cell number could be observed (34, 35). Furthermore, MM cells are able to directly target T cells by Fas/Fas ligand interaction (36).

Dendritic cells are antigen-presenting cells (APC) responsible for the induction of T cell responses. Although controversy remains about the number of DC in MM patients, it has been clear that they are functionally impaired. They show a lower expression of human leukocyte antigen (HLA) molecules and a reduced capacity in stimulating T cell proliferation and cytokine production (37). Furthermore, MM cells inhibit DC function by secretion of distinct cytokines including IL-6, IL-10, and TGF-β (38, 39).

Natural killer T cells (NKT) belong to the innate immune system and show important defects in MM patients. They not only decrease in cell number at the end-stage of the disease but it has been hypothesized that tumor-derived glycolipids also lead to a deficiency in IFNγ production and NKT dysfunction (40, 41).

Immune responses are also hampered by immunosuppressive cell types including regulatory T cells and TAM. TAM (CD68^+^) are increased in the BM of MM patients and promote cancer cell proliferation, angiogenesis, and drug resistance. In addition, TAM show a poor antigen-presenting capacity and inhibit T cell proliferation (42). Increased frequencies of regulatory T cells (CD4^+^CD25^high^FoxP3^+^) could also be observed in peripheral blood and BM of MM patients (43).

The development of immune modulating agents (IMiDs) including thalidomide, and its more potent and less toxic derivatives lenalidomide and pomalidomide, have made an important impact on treatment of relapsed/refractory MM patients (44). The exact molecular mechanism of IMiDs is not fully understood but broadly they affect the immune response by increasing T cell proliferation, NK cell-mediated lysis, and reducing regulatory T cells. Furthermore, IMiDs decrease bone resorption and angiogenesis (45, 46). Cellular immunotherapy, which is based on a graft-versus-myeloma effect (e.g., donor lymphocyte infusions, dendritic cell vaccines) showed promising results if utilized for relapsed or persistent MM after allogeneic stem cell transplantation (SCT) (47). However, immunosuppressive cell types limit the effectiveness of immunotherapy, indicating the importance of regulatory T cell, TAM and MDSC targeting in combination with current anti-myeloma drugs.

### MDSC characterization and distribution in MM

In our lab, MDSC were investigated using the 5T2 and 5T33MM mouse models. These models are derived from elderly C57BL/KaLwRij mice that spontaneously developed MM and are maintained by intravenous transfer of cancer cells into young syngeneic mice. Both models are immunocompetent and resemble the human disease closely as MM cells are able to grow in the surrounding BM microenvironment. The 5T33MM model has a more rapid growth (3 weeks), while the 5T2MM model is characterized by a moderate growth (12 weeks) and osteolytic bone lesions (48). In the BM, a clear shift toward a CD11b^+^Ly6G^low^ population, typically considered to be the phenotype of MO-MDSC, was observed at the end-stage of the disease in the 5T2 and 5T33MM mice (10). In addition, in this MO-MDSC population, we could discriminate different MDSC subsets based on differential Ly6C expression: inflammatory monocytes (Ly6C^high^SSC^low^), eosinophils (Ly6C^intermediate^SSC^high^), and immature myeloid cells (Ly6C^intermediate^SSC^low^). All of these populations may contribute to T cell suppression, with the monocytes being the most potent. Especially, a major increase in immature myeloid cells could be observed in the BM of the 5T33MM mouse model, indicating a differentiation block in the presence of MM cells (10). Of note, circulating CD11b^+^ cells increased only at the end-stage of the disease (data unpublished). In the similar 5TGM1 model, MDSC expansion in the blood, BM and spleen could also be observed up to 28 days after MM cell inoculation (21). In another immunocompetent mouse model, tumor cell lines derived from transgenic Bcl-xl/Myc mice (ATLN and DP42) were intravenously injected into syngeneic mice (22). A clear increase in BM MDSC (CD11b^+^GR1^+^) was shown the first week after MM cell inoculation with a similar increase in both MO-MDSC and G-MDSC subsets. The BM is the primary tumor site for MM cells and also the site where MDSC are generated. The direct contact of the cancer cells with myeloid progenitor cells might explain the early MDSC conversion and accumulation. During disease progression, MM cells take over the BM microenvironment and disturb and replace normal hematopoiesis. Therefore, the absolute number of MDSC in the BM initially increases, but then gradually decreases at later stages of disease, as observed in the 5T33MM model (data unpublished) and in the ATLN and DP42 models (22).

Some controversy exists about the MDSC characterization markers in MM patients. Brimnes and colleagues were the first to describe an increase of CD14^+^HLA-DR^−/low^ MO-MDSC in peripheral blood of MM patients at diagnosis compared to healthy donors (23). In contrast, recent studies demonstrated no difference in the MO-MDSC population, but a significant increase in G-MDSC, defined as CD11b^+^CD14^−^CD33^+^CD15^+^ (22) or CD11b^+^CD14^−^CD33^+^CD15^+^HLA-DR^low^ (12), in BM and peripheral blood of MM patients. Different flow cytometry analyses, limited patient numbers and distinct treatment regimens of MM patients could explain the discrepancy of the results (Table 1).

### MDSC expansion and mechanisms in MM

Little is known about the mechanisms of MDSC expansion, differentiation, and activation in MM. Although no specific studies in MM are conducted, distinct factors present in the MM microenvironment or secreted by MM cells are able to regulate MDSC expansion and activation. Possible factors include IL-6, GM-CSF, VEGF, and IL1β, which are mainly secreted by the myeloma BM microenvironment (49–51). STAT3 has been identified as the main transcription factor for MDSC expansion in distinct cancer models and the expression of the STAT3 target genes cyclin D1, MYC, survivin, and Bcl-xL, resulted in increased survival and proliferation. In addition, the myeloid-related proteins Arginase-1, S100A8, and S100A9 are upregulated by STAT3 (8, 52). S100A9 knockout (KO) mice showed a decrease in immature myeloid cells and a potent anti-tumor immune response (53). Importantly, a delay in the development of ovalbumin-expressing MM tumors was observed in S100A9KO mice compared to wild-type (WT) mice. Adoptive transfer of MDSC derived from WT-MM-bearing mice into S100A9KO MM mice resulted in a reduced survival (22), clearly demonstrating the importance of MDSC-derived S100A9 in MM (Figure 1B).

### Immunosuppressive function of MDSC subsets in MM

In the 5TMM as well as in the ATLN model, MDSC derived from MM-bearing mice showed clear immunosuppressive activity compared to immature myeloid cells from naïve mice (10, 22). In the 5TMM model, MO-MDSC were more potent to inhibit T cell proliferation compared to G-MDSC. Different subsets in the MO-MDSC population (inflammatory monocytes, eosinophils, and immature myeloid cells) contribute to immunosupression, with inflammatory monocytes being described as the most potent inhibitors. Moreover, myeloma-derived MDSC showed an upregulation of iNOS, arginase-1, and IL-10, compared to MDSC derived from naïve mice. The immunosuppressive action of MO-MDSC was partially abrogated by inhibition of iNOS by l-NMMA, while an arginase-1 inhibitor (norNOHA) did not affect T cell proliferation (10) (Figure 1A).

In human experiments, both MO-MDSC (CD11b^+^CD14^+^HLA-DR^−/low^) and G-MDSC (CD11b^+^CD14^−^CD33^+^CD15^+^) were sorted from BM of MM patients and significantly suppressed T cell activity *in vitro*. In contrast, immature myeloid cells derived from healthy donors did not exert any immunosuppressive activity (22). Görgün et al. described strong suppressive activities of MDSC on CD8^+^ T cells and NKT. As a control, they demonstrated that APC (CD11b^+^CD14^+^HLA-DR^+^) were able to increase T cell proliferation *in vitro*. However, this was in contrast with a recent study where both CD14^+^HLA-DR^low^ and CD14^+^HLA-DR^high^ cells derived from MM patients exerted immunosuppressive capacities (54). As observed in mice, specific inhibitors of arginase-1 and iNOS partially abrogated the immunosuppressive function of human MDSC (12).

### MDSC as osteoclast progenitors in MM

Besides their role in immune suppression, MDSC also play a pivotal role in bone disease. MDSC are macrophage progenitors which are able to differentiate into osteoclasts. MDSC derived from myeloma-bearing mice had a higher potential to differentiate into mature and functional osteoclasts *in vitro* and *in vivo* compared to MDSC from control mice. Co-inoculation of 5TGM1 MM cells and MDSC resulted in increased tumor burden and bone lesions. In addition, *in vivo* treatment with zoledronic acid, a potent nitrogen-containing bisphophonate, was able to induce a 30% reduction in MDSC (CD11b^+^GR1^+^) number, associated with a decrease in osteoclastogenesis to control levels (21). Interestingly, it has been shown that not all MDSC populations were able to differentiate into osteoclasts. Sawant et al. studied breast cancer MDSC derived from lung, blood, spleen, and lymph nodes and observed no osteoclastogenesis when cells were derived from these organs. However, BM MDSC from tumor-bearing mice underwent osteoclast differentiation in contrast to BM MDSC of naïve mice. Although factors responsible for this phenomenon need to be identified, a variety of osteoclastogenic growth factors including RANTES and MCP-1 are secreted by breast cancer cells (55). Although the early idea of MDSC is that they are blocked in their differentiation potential, it seems that in cancers involving bone disease, MDSC can differentiate into osteoclasts.

## MDSC in Lymphoma

### MDSC characterization and distribution in lymphoma

Lymphoma originates in the lymphatic system and is characterized by abnormal proliferation of B cells and T cells, mostly classified in Hodgkin and non-Hodgkin lymphoma. EG7 and EL4 are two well-characterized subcutaneous lymphoma models that are frequently used to investigate the MDSC subpopulations and functions. MO-MDSC (Ly6G^−^SSC^low^) and G-MDSC (Ly6G^+^SSC^high^) accumulated equally in the spleen of EL4 and EG7 murine models (5, 6). Furthermore, the majority of Ly6G^−^ cells showed increased F4/80 expression. Interestingly, three markers were differentially expressed in naïve and tumor-induced monocytes including CD71, CD115, and CD80, indicating a distinct MDSC phenotype in tumor-bearing mice compared to naïve mice (5, 6). Shlecker et al. investigated MDSC distribution in RMA-S lymphoma-bearing mice and found that MO-MDSC as well as G-MDSC accumulated in blood, spleen, and tumor tissue (56).

Little is known about the presence and characteristics of MDSC in human lymphoma patients. In B-cell non-Hodgkin lymphoma (NHL), peripheral blood mononuclear cells (PBMC) showed a reduced Th1-response as determined by IFNγ production compared to healthy controls. Furthermore, less T cell proliferation was observed after coincubation of PBMC with monocytes derived from NHL patients. Importantly, monocyte depletion by anti-CD14 immunomagnetic beads resulted in restored T cell proliferation. It has been shown that NHL monocytes had impaired STAT1 phosphorylation and IFNα production upon CpG oligodeoxynucleotides stimulation and defects in dendritic cell differentiation. No difference in the percentage of monocytes in peripheral blood of NHL patients could be detected compared to healthy controls; however, a clear shift in HLA-DR expression was observed. CD14^+^ monocytes in NHL patients showed a significant decrease in HLA-DR expression, which was correlated with suppressed immune functions and a more aggressive disease. In addition, elevated arginase-1 levels could be detected in plasma of NHL patients. Furthermore, NHL PBMC proliferation was increased by exogenous l-arginine administration *in vitro*, indicating that the arginine metabolism is at least partially responsible for the immunosuppressive effects (14) (Table 1).

### MDSC expansion and differentiation in lymphoma

Different factors contribute to MDSC expansion and differentiation in lymphoma models. Adiponectin has been identified as an important regulator of MDSC expansion in EL4 T cell lymphoma-bearing mice. Tumor-bearing adiponectin knockout (APNKO) mice showed reduced levels of splenic MDSC compared to WT mice and a higher amount of NK and CD8^+^ T cells. These effects were associated with reduced lymphoma growth in APNKO mice. In addition, this study demonstrated that G-CSF was lower expressed in tumor-bearing APNKO mice and that this factor played a key role in MDSC differentiation (57). Dardalhon and colleagues described a role for the Tim3-Galectin-9 pathway in MDSC proliferation. EL4 mice treated with anti-Tim3 antibodies showed a delayed tumor progression and a lower frequency of CD11b^+^GR1^+^ cells. The authors hypothesized that Tim3 on IFNγ-secreting T cells interact with galectin-9 expressed by MDSC and that this ligand/receptor interaction is involved in MDSC expansion and function (58). Interestingly, galectins have been identified as important modulators of monocyte and macrophage function. Galectin-1 positively correlated with the M2 macrophage marker CD163, while galectin-3 was highly secreted by activated M2 macrophages (59, 60). TAM predict a poor clinical outcome in classical Hodgkin’s lymphoma patients and galectin-1 has been identified as a potential biomarker for relapsed/refractory disease (60, 61).

Besides the ability of immature myeloid cells to rapidly differentiate into macrophages and DC *in vitro*, Youn and colleagues observed a preferential differentiation of MO-MDSC toward G-MDSC in EL4 tumor-bearing mice (62). MO-MDSC acquire morphological, phenotypical, and functional features of G-MDSC in tumor-bearing mice including a high ROS production and myeloperoxidase activity. Acquisition of the granulocytic phenotype was mediated by epigenetic silencing of the retinoblastoma gene by HDAC2. Schlecker et al. characterized tumor-infiltrating MDSC subpopulations in the RMA-S T cell lymphoma model. Interestingly, tumor-derived MO-MDSC showed increased levels of CCR5 ligands (CCL3, CCL4, and CCL5), which were also associated with regulatory T cell recruitment (56). Serafini and colleagues demonstrated a role for MDSC in regulatory T cell proliferation in the A20 B-cell lymphoma mouse model. They characterized MDSC as APC, which were able to expand regulatory T cells (FOXP3^+^CD4^+^) from a preexisting regulatory pool and not by T cell conversion. Furthermore, they demonstrated IL4Rα is expressed on MDSC and correlates with tumor progression. *In vivo* treatment with sildenafil reduced regulatory T cell expansion and prevented T cell anergy (63).

As observed in MM models, S100A9 protein has been described as an important regulator of MDSC expansion. Tumor-derived conditioned medium induced accumulation of MDSC and reduced dendritic cell differentiation. This was accompanied by increased S100A8 and S100A9 expression. S100A9KO mice injected with EL4 lymphoma cells resulted in a smaller tumor size or even tumor rejection. T cells derived from S100A9KO mice showed higher cytotoxicity against EL4 compared to T cells derived from WT mice. In addition, S100A9 overexpression in hematopoietic stem cells resulted in reduced dendritic cell and macrophage differentiation and accumulation of immature myeloid cells (53). Kälberg et al. demonstrated that the interaction between S100A9 and toll like receptor 4 (TLR4) promoted tumor growth (64). Quinoline-3-carboxamides or Q compounds (e.g., Tasquinimod) were able to block this interaction and inhibited tumor proliferation (65).

Recently, it has been demonstrated that accumulation of MDSC in tumor-bearing EL4 mice was not caused by increased survival of these cells. As a matter of fact, MDSC in tumor-bearing mice have a shorter lifespan than monocytes and neutrophils, but are rapidly replaced by new cells as determined by *in vivo* BrdU labeling and apoptosis assays. ER stress, present in tumor-bearing mice, causes TNF-related apoptosis-induced ligand receptors (TRAIL-R) upregulation in MDSC. The expression of DR5, a TRAIL-R, was significantly higher in MDSC derived from tumor-bearing mice compared to control mice. Furthermore, MDSC derived from DR5 KO mice showed increased survival compared to WT MDSC. Data clearly demonstrated that inhibition of DR5 improved CD8^+^ T cell responses in mice bearing TRAIL-insensitive tumors. For cancer patients, a decrease in survival of G-MDSC compared to granulocytic cells was observed. In addition, agonistic DR5 antibodies as well as TRAIL-recombinant protein was able to induce apoptosis of G-MDSC. Although no difference in DR5 expression between G-MDSC and granulocytic cells could be observed in patients, a lower expression of TRAIL decoy receptors 1 and 2 was determined (66) (Figure 1B).

### Immunosuppressive function of MDSC subsets in lymphoma

Different lymphoma models have been employed to study MDSC biology. The advantage of the immunogenic mouse BW-Sp3 thymoma is the existence of tumor progressors and regressors within the same model. The majority of the mice develop a tumor-specific CD8^+^ T cell response resulting in tumor regression but a significant fraction of the tumors ultimately start to progress. By studying mice with different tumor outcomes, a significant contribution of splenic CD11b^+^ GR1^+^ cells to the immune dysregulation seen in tumor progressors could be demonstrated. Indeed, while T-cell activating APC were induced in the spleen of regressors, T cell suppressive MDSC were increased in the spleen of progressors. These MDSC, in particular the MO-MDSC fraction, could differentiate into macrophages that had very high arginase levels and expressed high levels of M2-associated genes, suggesting that MDSC could be precursors of M2-oriented and immunosuppressive macrophages (15, 67). Interestingly, stimulation of the nuclear hormone receptor PPARγ, one of the M2-associated markers, diminished the suppressive potential of these MDSC-derived macrophages (15).

Also MO-MDSC and G-MDSC derived from EL4 and EG7 lymphoma models demonstrated immunosuppressive capacity *in vitro* (5, 6). While G-MDSC showed a higher ROS production, MO-MDSC had increased expression of NO and nitrotyrosine. l-NMMA (NO inhibitor) and arginase inhibitors were able to partially reverse MO-MDSC suppression. Suppressive activities of G-MDSC could be diminished by catalase, a hydroxyl peroxide inhibitor. Employing mice deficient for IFNγR or for the transcription factors downstream of IFNγR – STAT1 and IRF-1, it was demonstrated that EG7 lymphoma-induced MO-MDSC needed to be stimulated by IFNγ to become suppressive and that the suppressive mechanism was mediated by parallel IRF-1/iNOS-dependent and IRF-1/iNOS-independent pathways (68). G-MDSC only minimally depended on IFNγ and iNOS for their suppressive activity (68). Kusmartsev et al. investigated the induction of T cell tolerance by immature myeloid cells from lymphoma-bearing mice *in vivo* (69). They adoptively transferred transgenic T cells specific for OVA-derived peptide and immature myeloid cells from EG7 tumor-bearing mice into naïve mice. Afterward, lymph node cells were isolated and they observed a dramatically reduced or eliminated antigen-specific T cell response after adoptive transfer of immature myeloid cells. Importantly, GR1^+^ cells from naive mice and DC from EL4 tumor-bearing mice were not able to inhibit T cell responses. In addition, it has been demonstrated that immature myeloid cells take up soluble protein *in vivo*, process the protein, and present antigenic epitopes on their surface to induce T cell anergy. Nagaraj et al. subsequently demonstrated that the incubation of MDSC with Ag-specific CD8^+^ T cells caused nitration of the molecules on the surface of CD8^+^ T cells that are localized to the site of physical interaction between MDSC and T cells. This process induces CD8^+^ T cell tolerance that is only specific for the peptide presented by the MDSC. MDSC caused dissociation between the antigen-specific TCR and CD3zeta molecules, disrupting TCR complexes on T cells (70). This is in accordance with the fact that EG7-derived MDSC decreased antigen-driven CD8^+^ T cell proliferation *in vitro*, while anti-CD3-driven proliferation was not affected, again indicating an antigen-specific suppression. Our own studies demonstrated that EG7 lymphoma-induced MDSC intricately influence different CD8^+^ T cell activation events, whereby some parameters are suppressed while others are stimulated (68). For example, while CD8^+^ T cell proliferative capacity and IL-2 secretion are clearly diminished in the presence of MDSC, the IFNγ production by these cells is actually stimulated on a per cell basis. Complex effects of MO- and G-MDSC on CD8^+^ T cell adhesiveness to the extracellular matrix and to selectins, on sensitivity to FasL-mediated apoptosis and on cytotoxicity were also noted (Figure 1A).

## MDSC in Leukemia

Leukemia is characterized by abnormal proliferation of immature white blood cells that usually starts in the BM. Four main types of leukemia are defined: acute myeloid leukemia (AML), chronic myeloid leukemia (CML), acute lymphoblastic leukemia (ALM), and chronic lymphocytic leukemia (CLL). Studies on MDSC in leukemia are limited. At diagnosis, peripheral blood of AML patients showed a high heterogeneity in MDSC percentage (CD14^−^HLA-DR^−^CD33^+^CD11b^+^) and a negative correlation with CD34 percentage (24). Christiansson et al. demonstrated increased MDSC numbers, phenotyped by CD11b^+^CD14^−^CD33^+^, and arginase-1 expression in CML patients (25). In untreated CLL patients, the circulating CD14^+^HLA-DR^low^ cells were increased compared to healthy controls. In addition, these cells were positive for myeloid markers (CD33, CD11b, CD13, CD11c) and expressed the macrophage colony-stimulating factor receptor CD115 and IL4α receptor, which are associated with MDSC activity. MDSC suppressive capacity *in vitro* was demonstrated and linked to indoleamine 2,3-dioxygenase (IDO) expression. Furthermore, MDSC promoted regulatory T cell development indicating a cross-talk between CLL cells, MDSC, and regulatory T cells (13). MDSC markers described in literature for MM, lymphoma, and leukemia are summarized in Table 1.

Besides the observations illustrating the importance of MDSC and regulatory T cells in T cell tolerance, it has to be remarked that T cell dysfunction can also be independent of these immunosuppressive cell types. Zhang and colleagues observed T cell dysfunction in a murine model for AML, which was antigen-specific and could not be reversed after MDSC or regulatory T cell depletion. It has been argued that T cell tolerance in that case is regulated by tolerogenic APC. Consequently, T cell activation and prolonged survival could be achieved by the use of agonistic anti-CD40 antibodies, which induces immunogenic DC (71).

## MDSC Targeting Strategies in Hematological Malignancies

MDSC could be considered as a valid therapeutic target since they contribute to distinct processes in tumor development, progression and metastasis (18, 72). MDSC targeting can be achieved by distinct strategies including MDSC depletion, MDSC deactivation, induction of maturation/differentiation, and a block in MDSC development (18, 73). Currently used MDSC targeting drugs are listed in Table 2.

**Table 2:** **General overview of MDSC targeting agents**.

Targeting strategy	Mechanims of action	Example(s)	Reference
MDSC deactivation	Phosphodiesterase (PDE5) inhibitors	Tadalafil Sildenafil	(74–76)
	NO inhibitors	l-NAME	(10, 12, 77)
		Nitroaspirin	
	COX2 inhibitors	Celecoxib	(78)
	Arginase inhibitors	NOHA	(10, 12)
		l-NAME	
	ROS inhibitors	Synthetic triterpenoids (e.g., Bardoxolone methyl)	(79)
	IDO inhibitors	d,l-1-methyl-tryptophan	(80)
MDSC depletion	Cytotoxic agents	5-Fluorouracil Gemcitabine	(81)
	HSP90 inhibitors	17-DMAG	(82)
	Peptibodies	Peptide-Fc fusion proteins	(83)
Induction of MDSC differentiation	Vitamins	ATRA Vitamin A Vitamin D3 Vitamin E	(69, 84–86)
	Antibodies	GR1 antibodies	(87, 88)
Block in MDSC development	N-Bisphophonates	Zoledronic acid	(21)
	Multi-kinase inhibitors	Sunitinib Sorafenib	(89, 90)
	JAK2/STAT3 inhibitors	Cucurbitacin B JSI-124	(91, 92)
	Blocking antibodies	Anti IL-17 antibodies Anti-glycan antibodies	(93–95)
	Quinoline-3-carboxamide derivative	Tasquinimod	(64, 96)

*NO, nitric oxide; COX2, cyclooxygenase-2; NOHA, N-hydroxy-l-Arginine; L-NAME, N(G)-nitro-l-arginine methyl ester; ROS, reactive oxygen species; IDO, indoleamine 2,3-dioxygenase; HSP90, heat shock protein 90; 17-DMAG, 17-dimethylaminoethylamino-17-demethoxygeldanamycin; ATRA, all-*trans*retinoic acid*.

### MDSC deactivation

MDSC suppressive mechanisms are often associated with the l-arginine metabolism, which has been extensively reviewed (8, 18, 77). Activated MDSC express high amounts of arginase-1 and NOS2, and inhibitors of both enzymes (L-NMMA for NOS2 and norNOHA for arginase-1) reversed MDSC suppressive mechanisms in MM and lymphoma models (10, 12, 68). Phosphodiesterase (PDE5) inhibitors (such as Sildenafil) inhibit cyclic guanosine monophosphate (cGMP) degradation and reduce arginase-1 and NOS2 expression. It has been hypothesized that high levels of cGMP interfere with the IL4Rα expression by MDSC, resulting in reduced levels of arginase-1 and NOS2. Consequently, Sildenafil treatment of peripheral blood mononuclear cells isolated from MM patients resulted in increased T cell proliferation *in vitro* (74). Another PDE5 inhibitor, Tadalafil, entered a phase II clinical trial to improve the response to dexamethasone and lenalidomide in 13 MM patients who were refractory to lenalidomide. However, the study was stopped due to a lack of response (75). Recently, the group of Borello et al. demonstrated a reduction in M-spike by Tadalafil treatment in an end-stage relapsed/refractory MM patient. BM CD14^+^ cells decreased over time by Tadalafil treatment. This was associated with a decrease in IL4Rα, iNOS, and arginase-1 expression in MDSC. BM nitrosylation was also decreased and T cell activity enhanced upon Tadalafil administration (76).

The tryptophan-degrading enzyme IDO is synthesized by MDSC and contributes to immune tolerance by mediating T cell suppression. IDO locally depletes tryptophan and generates tryptophan metabolites including kynurenine resulting in reduced proliferation of CD4^+^ T cells, CD8^+^ T cells, and NK cells (97). IDO activity has been correlated with increased immunosuppressive activity in MM patients and can be targeted by d,l-1-methyl-tryptophan (80). ROS secretion also contributes to the immunosuppressive action of MDSC and is caused by the increase in NADPH oxidase activity in granulocytic MDSC. Increased ROS levels were observed in EL4 tumor-bearing mice and could be targeted by a STAT3 inhibitor JSI-124 (91). Synthetic triterpenoid Bardoxolone Methyl (CDDO-Me) also reduced ROS and nitrotyrosine levels in EL4 mice, which was accompanied by an increased T cell response and reduced tumor load (79). Although not tested in hematological malignancies, cyclooxygenase-2 (COX2) inhibitors have also been described to reduce MDSC numbers and their immunosuppressive function in mesothelioma (78).

### MDSC depletion

The MDSC-depleting capacity of cytotoxic agents including 5-fluorouracil (5FU) and gemcitabine has been explored in the EL4 murine lymphoma model (81). Both agents were able to deplete MDSC in the spleen and tumor bed, with 5FU being the most potent drug. Similar effects on granulocytic and monocytic subpopulations could be observed. It has been demonstrated that 5FU exerted anti-tumor activities at least in part by elimination of MDSC and the restoration of T cell specific immune responses. Another compound, the HSP90 inhibitor 17-DMAG, was recently tested in a murine sarcoma model and resulted in a reduced number of MDSC and regulatory T cells upon *in vivo* administration; however, the underlying mechanism remains unknown (82).

Recently, peptide-Fc fusion proteins, named peptibodies, were developed to target the MDSC population *in vivo*. Via a competitive phage display platform MDSC specific peptibodies were identified. The peptibodies were engineered to express the mouse IgG2b Fc portion to enhance antibody-dependent cell-mediated cytotoxicity (ADCC). Intravenous peptibody injection was able to deplete blood, spleen, and intratumoral MDSC in distinct lymphoma models (A20, EG7, EL4). No effects on other immune cells, including DC and lymphocytes (T, B, and NK cells), could be detected. Importantly, peptibody treatment in EL4 tumor-bearing mice delayed tumor growth *in vivo* as determined by tumor size and tumor mass. In contrast to anti-GR1 specific antibodies, which predominantly target the granulocytic population, peptibodies were able to deplete both monocytic and granulocytic MDSC (83).

### Induction of MDSC differentiation

Another mechanism to target the MDSC population is the induction of MDSC differentiation into mature myeloid cells with no suppressive activities. MDSC differentiation can be triggered by distinct vitamins including vitamin A, vitamin D3, or vitamin E (84–86, 98). ATRA (all-*trans*retinoic acid), a vitamin A metabolite, induces the differentiation of monocytic MDSC in DC and macrophages, and causes apopotosis of the granulocytic MDSC population. As a consequence, ATRA improved immunotherapy in distinct murine models (98).

Anti-GR1 antibodies are described to bind with a high affinity to Ly6G molecules and have been extensively used to deplete G-MDSC in tumor-bearing mice. In contrast, Ribechini et al. demonstrated that anti-GR1 antibodies induced myeloid expansion and upregulation of macrophage markers (F4/80, CD115) in BM-derived MDSC. In addition, they observed increased STAT phosphorylation (STAT1, STAT3, STAT5) by anti-GR1 antibody treatment of BM cells *in vitro*. Protection of BM MO-MDSC against apoptosis was correlated with increased expression of the anti-apoptotic factor Mcl1. Despite the incapacity of MDSC depletion, anti-GR1 antibodies transiently decreased the immune-suppressive activity of GR1^high^ and GR1^low^ subpopulations (87). The effect of anti-GR1 antibodies was also investigated in EL4 tumor-bearing mice (88). In this murine lymphoma model, a complete elimination of MDSC in the spleen and peripheral blood was observed, but hepatic MDSC remained. Despite induction of apoptosis of hepatic MDSC by anti-GR1 antibodies, the population is immediately replaced and showed immunosuppressive activities.

### Block in MDSC development

IL-17 has been identified as an important factor in MDSC development. IL-17R^−/−^ EG7-OVA tumor-bearing mice (EL4 transfected with the chicken ovalbumine gene) showed a lower MDSC number compared to WT mice (93). In addition, reduced arginase-1, MMP9, and S100A8/9 expression was observed in IL-17R^−/−^ compared to WT MDSC. IL-17 neutralizing antibodies were able to decrease tumor growth in WT mice indicating the importance of IL-17 targeting strategies in cancer treatment (93). Besides the effect on MDSC expansion, it has been demonstrated that S100A8/S100A9 proteins are also involved in MDSC recruitment and retention. Migration to the tumor site is promoted by the binding of S100 proteins to carboxylated N-glycan receptors. The anti-carboxylated glycan antibody mAbGB3.1 was able to reduce MDSC numbers in the blood and secondary lymphoid organs (99). Furthermore, mAbGB3.1 was able to block tumor cell proliferation in colorectal cancer (94, 95). In addition, Tasquinimod, a quinoline-3-carboxamide derivative, binds to S100A9 and blocks the interaction with its ligands receptor of advanced glycation end products (RAGE) and toll like receptor 4 (TLR4). It has been demonstrated that Tasquinimod reduced MDSC accumulation, modulated local tumor immunity, and reduced tumor growth and metastasis (64, 96).

As previously mentioned, the *N*-bisphosphonate zoledronic acid reduced MDSC number and osteoclast formation in MM disease (21). JAK2/STAT3 inhibitors (JSI-124 and Cucurbitacin B) (91, 92) and multi-kinase inhibitors (Sunitinib and Sorafenib) were also described to reduce MDSC levels (89, 90). Cucurbitacin B induces differentiation and maturation of DC (92). Sunitinib treatment resulted in 50% reduction of peripheral blood MDSC, reduced regulatory T cell number, and enhanced Th1 response in renal cell cancer patients (89). Sorafenib, another multi-kinase inhibitor, decreased immunosuppressive cell types in hepatocellular carcinoma (90).

## MDSC in Hematopoietic Stem Cell Transplantation

Hematopoietic SCTs have been used as a frequent therapy in blood and BM cancers. However, infections and graft-versus-host disease (GVHD) remain a major cause of mortality. In order to reduce GVHD in allogeneic hematopoietic stem cell transplantation (HSCT), it might be of interest to expand the MDSC population and reduce allogeneic donor T cell activity in patients. Interestingly, it was found that MDSC numbers were higher in SHIP^−/−^ mice displaying a reduced GVHD compared to WT mice (100). In subsequent studies, it was demonstrated that administration of MDSC together with BM transplantation was able to significantly inhibit GVHD lethality and was associated with a decreased proliferation and activation of donor T cells (101, 102). Furthermore, arginase-1 is an important contributor to this effect since administration of a PEGylated form of Arginase-1 could reduce GVHD lethality. In preclinical models, it is described that during BM chimerism monocytic and polymorphonuclear MDSC subsets with alloreactive T cell suppressive capacity have expanded (103). Interestingly, phenotypical and functional studies demonstrated an expansion of similar subsets, MO-MDSC (CD14^+^CD15^−^) and G-MDSC (CD14^−^CD15^+^), in peripheral blood derived from G-CSF-treated stem cell donors (103). Furthermore, an increased frequency of T cell suppressive CD14^+^ HLA-DR^low/neg^ cells was found in allo-HSCT patients after transplantation (26) (Table 1). All these studies indicate that MDSC might have a beneficial role in preventing GVHD and thus can have important clinical implications.

## Perspectives

The past years, extensive research has been performed on MDSC in hematological malignancies. Despite the increasing knowledge, many questions remain concerning the role of distinct MDSC subtypes and mechanisms inducing MDSC expansion and/or survival. MDSC differentiation stages toward TAM and tumor-associated neutrophils also remain an unexplored field. In addition, caution in the interpretation of murine experiments is needed. Subcutaneous tumor models (including EG7, EL4, A20) have the major limitation of a localized tumor growth and are less representative because of the lack of an appropriate tumor microenvironment.

Immune modulating agents, especially in combination with other drugs, have significantly improved the outcome of patients with relapsed/refractory MM and lymphoma (104, 105). However, cancer-induced accumulation of immunosuppressive cell types including MDSC, TAM, and regulatory T cells counteract this immune reaction. In order to improve the efficacy of immune modulating drugs and to induce a durable anti-tumor immune response, MDSC targeting could be of great interest. Recently, Tadalafil, a PDE5-inhibitor, induced a long-term anti-myeloma immune and clinical response in a patient with end-stage relapsed/refractory MM (76). In addition, MM-induced osteolytic bone lesions could be reduced by MDSC targeting strategies. Currently, no specific MDSC targeting agents are available and hamper further investigation. Development of new agents and combination studies of MDSC targeting drugs (e.g. 5FU, anti-GR1) with currently used therapy for MM, lymphoma and leukemia are necessary in the future.

## Authors Contribution

Kim De Veirman, Els Van Valckenborgh, and Jo A. Van Ginderachter wrote and critically revised the paper. Elke De Bruyne, Eline Menu, Ivan Van Riet, Qods Lahmar, Xenia Geeraerts, and Karin Vanderkerken critically revised the paper.

## Conflict of Interest Statement

The authors declare that the research was conducted in the absence of any commercial or financial relationships that could be construed as a potential conflict of interest.
